# Lamotrigine Extraction and Quantification by UPLC-DAD in Plasma from Patients with Bipolar Disorder

**DOI:** 10.1155/2022/3288646

**Published:** 2022-04-13

**Authors:** Claudia V. Palacios-Magaña, Elba M. Romero-Tejeda, Nicté S. Fajardo-Robledo, Luis J. González-Ortiz, José G González-Mendez, Fermín P. Pacheco-Moisés

**Affiliations:** ^1^Departamento de Química, Universidad de Guadalajara. Boulevard Gral. Marcelino García, Barragán 1421, C.P. 44430, Guadalajara, Jalisco, Mexico; ^2^Departamento de Farmacobiología, Universidad de Guadalajara. Boulevard Gral. Marcelino García, Barragán 1421, C.P. 44430, Guadalajara, Jalisco, Mexico; ^3^Instituto Jaliscience de Salud Mental. Av, Zoquipan 1000, C.P. 45170, Zapopan, Jalisco, Mexico

## Abstract

A sensitive and efficient analytical process for detecting lamotrigine in acidic solution based in ultra-high-performance liquid chromatography-diode array detector (UPLC-DAD) was developed; the stationary phase used was a C8, 150 × 4.6 mm, 2.6 *µ*m. The mobile phase consisted of acetonitrile/acidified water (0.01% H_3_PO_4_ and 0.005% triethylamine, pH 2.4) (25 : 75 v/v). Limits of detection and quantification were 0.02 *µ*g/mL and 0.05 *µ*g/mL, respectively. The working interval for the evaluation of the method ranged from 0.05 to 12 *µ*g/mL, and the linear fit of the experimental data has a value of *r*2≥0.98. Before quantifying lamotrigine in plasma of patients with bipolar disorder, lamotrigine was released from plasma proteins with a 0.2 M sodium hydroxide solution, and then proteins were removed by precipitation with acetonitrile. Afterward, the lamotrigine base was dissolved in ethyl acetate. This extract was reconstituted in potassium phosphate solution (pH 2.4) to obtain more than 98% of lamotrigine protonated in N_2_, which was detected and quantified as indicated above. The absolute percentage of lamotrigine recovery is  ≥80% for all tested concentration levels. The accuracy and precision of the method have %CV values <4% for the lamotrigine levels of 3, 6, and 9 *µ*g/mL. The correlation coefficient for the used concentration range is 0.99. The analytical method is precise and sensitive to measure lamotrigine levels expected in plasma of BD patients and these levels were in the therapeutic dose range.

## 1. Introduction

Lamotrigine (3,5-diamino-6-(2,3-dichlorophenyl)-1,2,4-triazine) is a phenyltriazine-derivate used in the treatment of bipolar disorder (BD), a disease characterized by extreme changes in the mood [1]. BD is the seventh leading cause of disability worldwide, affecting 2.4% of the world's population and leading to suicide in 20% of the population suffering from it [2]. Lamotrigine is particularly effective in maintaining remission and preventing depressive episodes [3] and has received regulatory approval for its use in more than 30 countries, using the same doses as for convulsive epilepsy, that is, between 25 and 450 mg/day [1]. However, the dose of lamotrigine for use as a mood stabilizer in BD patients has not been validated. The broad therapeutic interval of the treatment makes it necessary to monitor the drug in human plasma, which can be used to assess its dose-response effects and know the specific pharmacokinetic profile in patients with BD.

Several analytical methods to assess lamotrigine in biological fluids have been described, such as gas chromatography, gas chromatography-mass spectrometry, radioimmunoassays, stripping voltammetry, electrospray ionization-mass spectrometry, capillary electrophoresis, and liquid chromatography [4]. For example, UHPLC analysis with a mass detector and treatment of the samples using protein precipitation has recently been described [5, 6]. The methods proposed by Ferreira et al. (2014) [7] and Ventura et al. (2014) [8] are relatively similar to those described by Serralheiro et al. (2013) [9], and despite being more reliable, they use expensive consumables such as hydrophobic reversed-phase C18 cartridges.

Essential factors to achieving efficient extraction and quantification of lamotrigine from human plasma are pH, protein precipitation method, and detection wavelength. Commonly, pH used for the extraction of lamotrigine in human plasma oscillates between 5.5 and 6.5, close to the value of pKa (5.89). In this condition, neutral and protonated forms in N_2_ coexist, which leads to a broadening of the chromatographic peak. The base form predominates at pH ≥ 8, and the protonated structure in N2 at pH <4 [8, 10]. The pH can be selected to favour the presence of a single ionic form of lamotrigine which can eliminate variations in the detection method caused by the coexistence of its different ionic forms. The used methods of protein precipitation can induce the loss of lamotrigine since 55% of lamotrigine is bound to plasma proteins [11]. Usually, the wavelength used for detection of lamotrigine, in the detection system, is very close to the wavelength of the solvents used in chromatography and is common for a large number of molecules [7, 9], which limits the selectivity of the method. In the method described by Asadi et al., [12] plasma proteins were precipitated with acetonitrile and subsequently resuspended in buffer at pH 8, which prevents ionization of lamotrigine and increases its liposolubility. However, the mobile phase used was a methanol/water (45 : 55, v/v). Greiner and Haen do not propose a lamotrigine extraction method, and in their quantification procedure, they use a cut-off wavelength similar to the cut-off wavelength of solvents. In addition, this method lacks specificity since the other two molecules have the same retention time of lamotrigine [13]. The limitations of these methods prompt us to develop a simple analytical method that is specific, sensitive, and accurate. Thus, the aims of this work are to develop a new efficient analytical process for (1) detection of only one of the ionic species of lamotrigine (protonated in N2) in acid solution based on UPLC-DAD; (2) extraction of lamotrigine from plasma proteins at alkaline pH, minimizing the loss of lamotrigine caused by the method of precipitation of proteins with organic solvents of medium polarity; and (3) to quantify lamotrigine in plasma of TB patients.

## 2. Materials and Methods

### 2.1. Chromatography Conditions

Lamotrigine extracts and solutions were evaluated in Agilent 1260 UPLC-DAD equipment. Analyses were carried out at a wavelength of detection of 266 nm and a C8 stationary phase, 150 × 4.6 mm, 2.6 µm (Phenomenex®). Mobile phase was acetonitrile/acidified water (0.01% H3PO4 and 0.005% triethylamine, pH 2.4) (25 : 75 v/v), with 0.5 mL/min flow, 20 µL injection volume, temperature 10°C, and column temperature 25°C. The total sample run time was 15 min.

### 2.2. Validation of the Analytical Process

The performance parameters were evaluated according to the Bioanalytical Method Validation Guidelines for Industry from the US Department of Health and Human Services, Food and Drug Administration [14].

### 2.3. Lamotrigine Extraction Method from Human Plasma

To 500 µL of plasma containing known concentrations of lamotrigine and plasma from BD patients treated with lamotrigine, 50 µL of 2M sodium hydroxide solution was added and vortexed for 30 s. Then, 2 mL of cold acetonitrile was added and immediately vortexed for five minutes and incubated at −20°C for 20 minutes. Afterward, the samples were centrifuged at 3400 rpm for 10 min; the organic phase was separated by decantation and centrifuged at 3400 rpm for 10 min. Again the supernatant was decanted and evaporated to dryness at 18 psi and 75°C for 60 min; the residue was resuspended in 2 mL of ethyl acetate, mixed by gentle inversion for 4 min and centrifuged at 3400 rpm for 10 min and decanted. The supernatant was evaporated to dryness as above, and the residue was resuspended with 0.5 mL of the mobile phase.

### 2.4. Biological

Blood samples were taken by venipuncture in tubes containing ethylene-diamine-tetra-acetic acid as an anticoagulant. The tubes were centrifuged at 2500 rpm for 15 minutes, and the plasma was recovered and stored at -70°C until use. Blood was obtained from healthy volunteers and patients were diagnosed with BD after signing the informed consent. The Research Ethics Committee of the Jalisco Institute of Mental Health approved this study with the number 198. The integrity and confidentiality of the volunteers and patients were maintained according to the updated Declaration of Helsinki (version 64th General Assembly, Fortaleza, Brazil, October 2013) [15] and to the “Regulation of the General Health Law on Health Research” April 2014 [16].

## 3. Results and Discussion

### 3.1. Validation Procedure

The validation procedure followed the Bioanalytical Method Validation Guidelines for Industry from the US Department of Health and Human Services, Food and Drug Administration [14], as follows:

#### 3.1.1. Specificity

Under the established chromatographic conditions, diluents, mobile phase, and blanks do not generate a response to the retention time of lamotrigine.

#### 3.1.2. System Precision

Lamotrigine solutions (6 µg/mL) were prepared in sextuplicate and analysed. The %CV obtained was <1.5%.

#### 3.1.3. Adequacy

**Figure 1:**
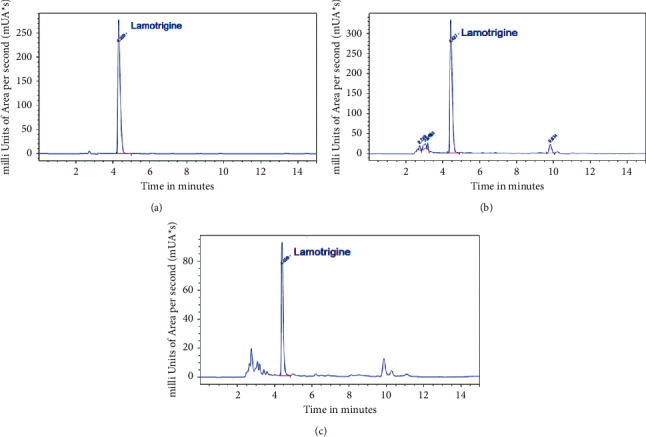
Chromatograms of lamotrigine in diluent (a), plasma extract loaded with lamotrigine (b), and plasma extract from a patient with bipolar disorder treated with lamotrigine (c).

A precision solution of lamotrigine was analysed five times. The %CV was ≤1.5%.  Figure 1(a) shows a typical chromatogram of lamotrigine in diluent.

#### 3.1.4. System Linearity

Five concentration levels of lamotrigine (1.6, 3, 6, 9, and 12 µg/mL) were prepared by dilution in triplicate. The relationship between concentration vs peak area was determined by the least-squares method. The calculated parameters were as follows: slope = 370.58; ordinate in the origin = -24.80, *r*2 = 0.99, and CI = 375.40–365.76.

#### 3.1.5. Stability

Convenient analysis steps were established to evaluate the stability of the lamotrigine standard solution and analytical solution. In the initial step, analytical stability was determined from the indicated homogeneous samples for each of the two different solution types, and samples were analysed in duplicate (initial analysis (ŷ0)) and stored. Then, samples were analysed within the specified storage times. The response of each sample was calculated: arithmetic mean of the initial analysis (ŷ0), arithmetic mean of the method (ŷ1), and absolute difference (|di|). Lamotrigine standard solution was stable for three months at 4°C. The analytical sample was stable for three days at 10°C. The plasma sample was stable for 12 hours at 4°C, for one month at -20°C, and for 12 months at -70°C, for all samples, |di|≤ 5.

#### 3.1.6. Limit of Detection and Quantification

The responses of blanks (reagents, plasma, mobile phase, and diluents) were made in sextuplicate. The limits of detection and quantification values were calculated at a signal-to-noise ratio of 3 and 10, respectively, and were 0.02 µg/mL and 0.05 µg/mL, respectively. The %CV was ≤10% for both limits.

#### 3.1.7. Linearity of the Method

3 concentration levels (1.6, 6, and 12 µg/mL) of analytes were prepared in triplicate, keeping the amount of placebo constant. They were analysed using lamotrigine standard solution as a reference. The recovered amount of analyte was determined. The parameters of the least-squares estimation method for the relationship between quantity added and quantity recovered were *b*1 = 0.75, *b*0 = 0.35, *r*2 = 0.99, IC(*β*1) = 0.8–0.7, IC (*β*0) = 0.69–0.006, and CVy/x < 5. The recovery percentage was >80%, standard deviation = 8, %CV <10, and ICµ = 80–94.

#### 3.1.8. Accuracy and Repeatability of the Method

Samples with analyte at six 6 µg/mL were prepared in sextuplicate. They were analysed using lamotrigine standard solution as a reference. The recovered amount of the analyte was determined. The recovery percentage of each analytical placebo was calculated by obtaining the quotient of the recovered quantity concerning the added quantity expressed in percentage. ŷ, S, CV, and IC (µ) of the recovery percentage were calculated. It was evaluated that IC (µ) included 80% or that the arithmetic average of the percentage of recovery was included in the interval 80 to 94% for this biological method and that the CV of the recovery percentage was not greater than <4%.

#### 3.1.9. Precision of the Method or Reproducibility

It was evaluated, preparing in triplicate plasma loaded at the concentration of the working range, on two different days and by two different analysts. The arithmetic mean ŷ, the standard deviation *S*, and the %CV were calculated. The %CV was <6%.

#### 3.1.10. Robustness

Instrumental factors (temperature of the autosampler, flow rate) and noninstrumental factors (pH of the phase, proportion of phases, proportions of solvents for extraction) were studied in each different operating condition and under normal conditions. The percentages of lamotrigine in the samples were calculated for both conditions. The arithmetic mean of the standard operating condition (ŷ0) and the arithmetic mean of each operating condition other than the standard one (ŷ1) were calculated, and the absolute difference of these ƖdiƖ was calculated.

### 3.2. Lamotrigine Extraction

As indicated previously, the analytical methods for the extraction of lamotrigine in human plasma are not suitable for the objectives of our project. Thus, based on the physicochemical properties of lamotrigine, the experimental procedure is as follows: lamotrigine was released from plasma proteins with a 0.2 M sodium hydroxide solution, which prevents the loss of protein-bound lamotrigine. Then, proteins were removed by precipitation with acetonitrile. Afterward, lamotrigine base was dissolved in ethyl acetate (that provides the ideal organic polarity to recover integrates the lamotrigine base molecule and thus separates it from hydrophilic components). This extract was reconstituted in acidic potassium phosphate solution (pH 2.4) to obtain more than 98% lamotrigine protonated in N2, which was detected and quantified by UPLC-DAD.  Figure 1(b) shows a typical chromatogram of the extracts of human plasma spiked with lamotrigine. With this experimental approach, the absolute percentage of lamotrigine recovery was >80% for all tested concentration levels. The accuracy and precision of the method have %CV values < 4% for the lamotrigine levels of 3, 6, and 9 µg/mL. The correlation coefficient for the concentration range is 0.99. The lamotrigine solution is stable for three months under refrigeration (4°C). The analytical samples are stable for one month stored at −20°C or −70°C. The plasma samples are stable for 12 hours at 4°C, for one month at −20°C, and for 12 months at −70°C.

Recently, Gangurde et al. [17] used a statistical experimental design (central composite design) for optimization of an HPLC method for detection of lamotrigine in lipid nanoformulations, in which the excipients are known. However, the human plasma is a more complex biological matrix and such method cannot be applied directly to analyze plasma from patients receiving treatment with lamotrigine. In addition, the physicochemical properties of lamotrigine must be taken into consideration for that purpose. Here, we developed an analytical method for lamotrigine extraction based on the physicochemical characteristics of lamotrigine in solution at acidic pH. The method is precise, is reliable in recovery and linearity, and allows us to obtain results in a short period of time. Furthermore, the method prevents the loss of lamotrigine from the biological matrix when proteins were removed with organic solvents [12].

### 3.3. Lamotrigine Levels in Plasma of BD Patients

Figure 1(c) shows a typical chromatogram of plasma extract from a patient with BD treated with lamotrigine and Table 1 shows the plasma levels of lamotrigine after 1, 3, 4, and 6 months of treatment. Analysis of variance showed that there is no significant difference in the average test scores across the time of treatment (*p* = 0.3184). These values were similar to those reported in the plasma of patients with several mood disorders [4] and serum of patients during the remission phase of acute bipolar II depression [18].

It is worth to note that the quantification of lamotrigine levels can be used to analyze the efficacy of the treatment according to the psychometric instruments used for assessment of symptoms associated with BD. We are currently developing experiments in the laboratory to assess the effect of lamotrigine treatment on several plasma markers of oxidative stress, with the evaluation of plasma lamotrigine levels being an aspect to consider in the evaluation.

## 4. Conclusions

The analytical method here developed is based on the physicochemical properties of lamotrigine, is sensitive to the concentrations of lamotrigine expected in plasma of BD patients, and follows international guidelines for bioanalytical methods. This method can be valuable in pharmacological and clinical monitoring research projects, whose benefits could include evaluating adverse effects, establishing individualization of drug dosage by therapeutic monitoring, and emphasizing the improvement of the pharmacological treatment of the disease.

## Figures and Tables

**Table 1:** Lamotrigine levels in plasma of BD patients treated at the dose of 200 mg/day.

	Mean ± SD
1 month (*n* = 7)	8.07 ± 3.71
3 months (*n* = 10)	8.05 ± 4.69
4 months (*n* = 11)	5.74 ± 2.76
6 months (*n* = 7)	5.30 ± 3.61

Lamotrigine levels were determined in BD patients after the start of the treatment.

## Data Availability

Data are available upon request from the corresponding author.
